# Madness, or the Maniac's Hall; a Poem, in Seven Cantos

**Published:** 1842-04

**Authors:** 


### Art. VIII.

*Madness, or the Maniac's Hall; a Poem, in Seven Cantos.* By the Author of the "Diary of a Solitaire."—*London,* 1841. 8vo, pp. 302.

Our critical labours seldom extend into the department of poetry, but the subject of the work before us brings it legitimately within our domain. The work itself demands notice as bearing the stamp of a powerful and elevated imagination, and of a highly-cultivated intellect, exercised under very peculiar circumstances, upon one of the most interesting topics within the range of medicine. Its history is singularly interesting, the poem having been wholly composed when the author was in the seclusion of a private lunatic asylum. From a letter written to the author by Mr. Southey, we transcribe the following passages, to which, for many reasons, much interest attaches itself:

"The subject is copious and important, but is it not of too exciting a nature for you? Your object should be what I proposed to myself as the one thing needful to intellectual self-contentment, five and thirty years ago, when I borrowed from an old Spaniard for my motto the words *in labore quies.* Any employment that agitates you must be so far injurious. Can you trust yourself for proceeding with it only while you find it beneficial, and lay it aside as soon as it affects you strongly? Long ago I was warned by experience never to proceed continuously with any work which I had in hand, after I had begun to dream of it; and this is the reason why I have always several works in progress."

We fear that this distinguished man overlooked a caution no less important than that which led him to withdraw from topics when they began to encroach on his good night's sleep; namely, that the brain will not bear continued labour, however varied, without exacting some penalty, sooner or later to be paid. If such labour is persevered in, the energies of mental life become prematurely exhausted, and the rest of intellectual existence is bound in shallows.

The deep and true feeling which animates the author of the poem is everywhere visible. His soul has evidently been sharply touched, but "to fine issues;" his destiny rough-hewed, but shaped to useful ends. In freedom, and in mental peace, which we trust he will long enjoy, and amidst the various comforts of social life, he retains the tenderest pity for those yet afflicted as he was once afflicted; and thus attempts in his own words,

> "The vivid transcript of those mournful days,
> When first this form, inured to madmen's ways,
> Was plunged within the breathings of dispair;
> Taught by the aid of sympathy's soft lays,
> The power to read of other hearts the care,
> And feel, e'en now, the chains which countless sufferers wear."

The poet begins by lamenting that other bards have left his subject unsung. He acknowledges the truth of Shakspeare's individual sketches, but still says:

> "Nor he, nor Pope, nor Dryden, e'er reveal'd,
> In deathless verse the pangs that long combine,
> Those mourning ones from human ken conceal'd,
> To dole their weary hours, where days no pleasure yield."
>
> *Canto* I. *St.* 9.

None, he says, have touched upon those

"mansions drear,
That bore the emblem of an earthly hell,
Where sympathetic tear of relatives ne'er fell."
*Canto* I. *St.* 11.

Few or none indeed had the opportunity of doing so with any useful effect. To them, as to the public in those days, the moping idiot and the madman gay were but the objects of a passing curiosity. The chained lunatic was left to crown himself with straw, the monarch of a dreary solitude, broken only by the curious or frightened gaze of visitors, admitted perhaps on payment of a penny to see the collection, and who carried away with them doleful impressions of dirt and obscenity, and of strong *keepers* (the very term seems to have been borrowed from the lion-ward of the tower) armed with scourges. The scene is now changed; light has beamed into the maniac's cell, and the scourges have gone. A few heavy shadows may remain, but these also must soon disappear. Recollecting the fierce resistance of those who in the struggle out of which this light was evolved, preferred the darkness, we read with impressive interest the following stanzas in the author's second canto, touching on matters no less important than *Seclusion*, *The character of Attendants*, and *Restraint*. Let it be remembered that we have here no competitor for the fame of novelty, no advocate eager to be esteemed humane, no superintendent anxious for distinction, no private owner of a lunatic house desirous to consult his own interests. We have a witness from the grave of the lunatic's cell itself; one who has felt and known the results of what he reprobates, and whose testimony thus acquires a genuine and even a solemn interest.

IX.

Seclusion, then,—but plenteous streams of light
Pouring with radiant beam the window through,
Must mark thy castle fair; with groves bedight
And meads of softest green, and sprightly view
Of distant scenes;—a village spire or two,
And aught that can with sanity unite
A healthier look at life. Thus wisely do,
And well thy labours fortune shall requite
With well-earn'd gold; and time, the lost one's mental sight.

X.

But add to these—and seek them from afar—
Attendants on thy will, for task design'd
To watch and wait on all: * *
* * * *
But 'tis not selfish ones that e'er shall find
'Mid education's sons that fitting store
Of knowledge and of life with love entwined,
Which opes of memory's crowded cells each door,
By whom thou mayst a patient's long lost joys restore.
* * * *

XII.

He that with anger meets a madman's wrath,
Adds to the flame his judgment should subdue;
None, save a fool, would brave a lion's path,
When, 'mid the pangs of hunger, full in view
A heifer stands—but would that jaw eschew:

So when the rising flood of passion swells
And in the restless brain to mem'ry true
Each pictured scene some real suff'ring tells
To him not less than Eden's joys or fancy's hells;

XIII.

Choose then, oh thou, whose sovereign presence reigns
Amid the halls, the chambers, and the grounds
Of this thy chosen seat—him who nor chains
Nor force delights in ; but whose love surrounds
Each sorrowing one—and to the utmost bounds
Of prudent safety, would in mercy throw
The gentlest veil—and whilst a fool impounds
His victims dread in bondage and in woe,
He, to his aid would bid each milder accent flow.

*Canto* II.

It is proper, however, to inform the reader that the poet, near the conclusion of his notes (p. 299), concurs in an opinion expressed, unfortunately we think, by Mr. Tuke of York, "that there are cases in which, under the most favorable management, we should best consult the feelings of the patient, as well as the comfort of his companions, by the application of mechanical means of restraint." No opinion can be more hurtful and discouraging than this, in which we apprehend that the advantages ascribed to restraint are really due to the seclusion accompanying it; or if not, are believed in because the effects of a proper system of care and watchfulness, and the application of the various resources which ingenuity suggests to meet the difficulties of different cases, have not been witnessed. A means so sure to be abused as mechanical restraint should not be tolerated if not indispensable. The attempts hitherto made to prove it indispensable, even in particular instances, have certainly not been satisfactory. There are still asylums in which it is thought indispensable during the night *in every case.* It is the sole and grand resource of indolence.

There is a note appended to the stanzas above quoted (p. 219), in which the author says, that of all the subjects connected with the proper treatment of insanity, the importance of securing suitable attendants has most impressed itself upon his mind. "Nothing," he says, "is more calculated to disgust and increase the excitement of a convalescent and sensitive mind, than to be subject to the low and necessarily ignorant talk of even the most civil, and for their station in life the best informed of the class almost invariably chosen to perform the important duties of attendance and companionship." He adds, that "although unwilling to dwell much upon his own sufferings, he could a tale unfold of the horrors of being subject to the reckless, rude, and barbarous ignorance of attendants, that would make his readers shudder." Of this we entertain no doubt. In the best managed asylums, nothing but constant vigilance can prevent neglect or unkind treatment of the patients by some of the attendants. Some of them are always inclined to lapse into indolence, and this indolence is a fertile source of cruelty as well as of neglect.

The suffering inflicted on a sensitive lunatic, heretofore accustomed to good society, when he finds himself on his partial recovery limited to the companionship of a common attendant, but especially by the rude and jocose familiarity, or it may be the rougher behaviour of the low and ignorant keepers belonging to ill-conducted private lunatic establishments, is still more strongly alluded to in canto iv. The remuneration given to the upper attendants in the better-managed private houses is sufficient, one would think, to secure the services of young persons of education and attainments much superior to what are possessed by those now usually filling such situations; and we are convinced that many a governess and many a tutor, now pining in the solitary school-room of private families, distracted with spoiled children, and depressed by "the proud man's contumely," would be much more happily and even more usefully placed if they accepted such situations, the duties of which are by no means ignoble. Both parties would be benefited; the teachers, transformed into attendants, would enjoy more consideration, more liberty, more variety; and the patients consigned to their care would reap the advantage of their greater resources, their more varied conversation, and their accomplishments. The following stanzas point to the present evil arrangements in these respects, whilst they present a shadow of terrors which might be unfit for ampler description, and afford the relief of contrast by attracting the attention to an asylum in which the principles of humanity are kept constantly in view.

v.

Adjacent to proud London's ancient walls,
These limbs, to fearful custody consign'd,
Endur'd a conflict that the soul appals,
E'en in the lapse of twenty years;—nor mind
Of Herculean strength could, thus confin'd,
Re-breathe uninjur'd liberty's sweet air;
The vigorous frame, to ruffian hands assign'd,
In vain could aid the intellect's right care;
And bad to worse he came, while lingering sadly there.

vi.

What else could spring from chains and dungeons dire?

vii.

All vain the effort, to constrain by force
The impetuous mind, or stay its fierce career;
And conscious then that 'twere a vain resource,
'Mid anger's fitful tempests, thus to steer,
Some guardian angel, (ah! to memory dear!)
In mercy pointed to where Ouse's stream
With gentle current winds that terrace near,
On which Sol's earliest and resplendent beam
Shines o'er York's vale with peaceful ray and rest supreme.

viii.

Her various aid—the walk, the sight of all
That city, famed for art's refining grace,
Can give to him whose vagrant footsteps fall
Its streets and hills among;—and friends had place
In the attempt to gild the rich-fraught face
Of novelty.

*Canto* iv.

We confess that we think the kind of evidence we are now placing before the reader of more than ordinary importance; and we therefore give insertion to a *note* on the above passages, in which the author speaks with all the plainness of prose on the same subject:

"Whoever took sufficient interest in the painful subject of public and private asylums, to glance over the result of a parliamentary enquiry many years ago in the shape of voluminous evidence from medical and other gentlemen connected with these establishments, might have seen enough to shake the strongest nerve, and to raise many an aspiration of the deepest sympathy at the narration on oath of the abhorrent cruelties then inflicted upon the suffering and neglected inmates of such places as Old Bedlam, St. Luke's, and not a few private establishments, amongst which the one referred to in the text was included. It was mainly by the individual and persevering exertions of Samuel Tuke, of York, that the long indulged misdeeds of its county asylum were brought to light, and the present reformed state of things there secured. Acts of parliament, subsequent to the enquiry above referred to, have greatly changed the whole face of the subject; and owing to the salutary restraint under which all such places are kept by the periodical visits of appointed magistrates, it is scarcely possible that England can again go back to the worse than inquisition-like barbarities of thirty years ago. If the author of the present volume had not, after a life of strange and fearful vicissitude, arrived at the same point of feeling, and entire reliance on the immutable wisdom of Providence implied in that line of the poet Young,

'For all I bless thee, most for the severe,'

it would have been difficult for him to have recalled his own sufferings at B —— G —— house, without exciting more than ordinary indignation against its proprietors. But alas! the system, then all but unusual, would shield any enormities. Suffice it to say that the hand that now writes, with its fellow-hand, and with the feet, have been chained for days and weeks together in solitary confinement; and occasionally, also, by a longer chain, to a staple in the floor in the common day-room of some twenty or thirty other patients. So also, at night, have the metal handcuffs been used, where now under similar circumstances every species of coercion would be altogether abandoned.

"The utter want of classification, too, and the abhorrent scenes to which the eye was frequently witness in the treatment of other cases, rendered the chances of recovery tenfold greater [less]; but of course, as the history of nineteen asylums in twenty will prove, the longer a patient remained, the more profitable was he to the proprietor.

"The author, for several months, continued getting worse and worse; that is, more impatient and excited, and his friends were at length compelled to try a change; and at the Friends' Retreat at York, in less than a fortnight after his removal, he was restored, though his recovery was followed by a long period of fearful depression.

"He has often found it extort expressions of surprise, when, though rarely done, he has alluded to these past scenes of his painful history, to find that he so distinctly remembered the facts of which he was induced to speak. It is this circumstance, reader, that induces the author still to doubt whether his own case has always been in the ordinary sense of the word, altogether one of positive insanity. He is, however, willing to admit, that in that period of afflictive dispensation, an asylum was the most welcome as well as salutary retreat from the gaze of an unsympathising world.

"Should the present volume prove, by the blessing of Providence, in any degree the feeble instrument of attracting a more vivid and decided attention to the subject generally, the author will feel that his sufferings have not been permitted to befal him in vain. Assuredly, on this point of memory and self-cognizance of all that has passed, both at the house near London, at York, and at D ——, the author's recollection is, in the minutest points, clear and vivid in the extreme; although, since the former, more than twenty years have elapsed." (*Note*, p. 252.)

We have given this note without omissions, and several parts of it will give rise in many readers to obvious reflections, which we shall not therefore express. But the dreadful picture it gives of the habitual restraint that must have prevailed in an asylum in which so intelligent a patient as the author was so severely coerced, dwells forcibly on the mind. *The hands and the feet chained for days and weeks together!* or *a longer chain to a staple in the floor of the day-room!* Such were the substitutes for kind watching, or for simple and not unwelcome seclusion. The sensitiveness on the part of those labouring under impaired mind, and the strong recollection they preserve of much or all that is said and done in their presence, are, we fear, too little thought of. They are perhaps pretty generally admitted as existing, but they are forgotten when the insane are to be controlled.

In the following stanzas the author alludes to the value of well-timed medicine; most likely to be neglected, we apprehend, where the universal remedy of restraint is held in most esteem, and also to the important subjects of employment and amusements. On all these matters we cannot too often remind the reader that the poet speaks, not as a speculative theorist or benevolent enthusiast, (terms occasionally applied to the promoters of the humane system,) but as one who has *felt* the ills which he describes, and all the sweet and all the sorrowful influences of good and bad treatment.

XIV.

Nor yet, forgetful of that needful skill
Which health the body's waning strength requires,
Neglect not med'cine to provide; and fill
Thy stores with drugs and draught that rest inspires.
* * * *

XV.

* * But neither physic's aid
Nor diet treatment, thou mayst here enforce,
Will health restore, if maniacs are not made—
Aye, urged by means resistless—to walk the shade,
Or strike the bounding ball, or use the arms
In labours healthful, and with hoe or spade
Clear well the recreant weed, or fence from harms
The tender plant, and screen th' exotic's embryo charms.
* * * *

XVIII.

Next to the active walk, fail not to store
Thy table with amusements, such as men
May well indulge!—the chequer'd board—and floor
Mark'd with its points for bowling; and again
The level surface of the smooth green pen
In which the ivory globe with impulse sure
First strikes the red ball's martial side, and then
Each after other thus the balls endure
The well-urged blow, and reach the number'd sockets pure.

XIX.

Yes! soothing game, the "bagatelle board" hight,
Full many an hour of weariness and woe,
This anger'd mind, 'mid scenes of tempting light
Has spent in striking with no skilless blow
The errant ball of ivory—and so
Has 'scaped of ills a sadly lengthen'd train,
That else had bid its wearying moments flow
'Mong retrospections drear: thus then would fain
The maddening bosom seek a brief retreat from pain.

XX.

Nor, were the bard possessed of such retreat,
Should long the billiard-table wait his call.

XXII.

These various games, or such as these, we need,
To rouse the dormant sense, and stir its fires;
And though 'tis not in mortal hands to speed
A cure, 'tis well if sport some joy inspires:
And oh! how needful 'tis that hope conspires
With calmer patience to subdue the vein
Of passion's blood excited;—when retires
Sweet sympathy, and self is left to gain
Its bitter portion of defeat, distrust, and pain. *Canto* II.

The remarks of the poet, who is one of the Society of Friends, on religious services, and their effects on the insane, are very striking. There is scarcely any subject more embarrassing, we believe, to superintendents and chaplains than this. Sometimes the subject is treated of with too much enthusiasm by very well-meaning persons, and doubtless sometimes with too much apathy. But that public religious services are a source of comfort and consolation to some of the insane, and of at least innocent enjoyment to many, is unquestionable. It is yet too early to say how far and with what results religious attentions are available in particular instances. Nothing can determine this very important point but the careful observation of judicious chaplains after several years' experience. It is scarcely credible that in one of the great lunatic asylums in London there is said to be no chaplain; and that even a chapel has not yet been thought of; an asylum, too, in which there are many well-educated patients.

XXV.

'Tis not for bards to sing of creeds or forms,
Or mark the line which, in a house like this,
'Twere well to take—since real love conforms
To all that Heaven assigns—nor aught amiss
Deems he the pray'rful language, though not his,
Which in that house his gracious God prepares;
In truth of heart alone consists our bliss—
And Catholic or Quaker, this is theirs
Who join in peace and love th' accustom'd household prayers.

XXVII.

Oh! 'twere a sin surpassing all beside,
If 'mid the regions of distorted minds,
Nor prayer, nor hymn, to soften erring pride,
Rose on the memory which sorrow binds
In darkening chains, and madness only finds;—
If 'mid disease and sin's unconscious deeds
Hope dwelt not here—while through this vale man winds
His faltering way to death,—and silent pleads
His Saviour's blood, and there his glorious pardon reads. *Canto* II.

The author of the poem makes a feeling allusion in one part of it to the effects of music upon him; although with an apology to the respected shades of Fox and Penn, as music is among the things which the society discountenances. To expect permanent effects from musical sounds in the case of the insane would be no more reasonable than to look for durable impressions from it upon the sound mind; but it often soothes and sometimes diverts them; and in certain instances seems to create a sudden revulsion in the thoughts, insomuch that intractable patients will become silent when it is unexpectedly heard, and shed tears. The mention of it by the author in the following stanza is extremely interesting:

XXVII.

He only knoweth, Lord of heaven and earth,
How this wrapt soul with listening love hath wept
In secret praise to him who gave it birth;
And e'er, amid each threat'ning danger, kept
The wavering heart! And what, if overstept
The joys which thee, dear Fox, or noble PENN,
Would cause to start, and from such strains have leapt
Your prison-bounds! Oh! grant that, yet again,
Silence may soothe the spirit, nor ask the songs of men. *Canto* III.

We have been led to notice this work not as being perfect as a poetical composition, or as one of which all the views meet our approbation. We regard it as containing testimony to be set against the asserted anxiety of insane persons to be put in restraint, which has been one of the arguments used by those long habituated to the old methods of coercion. Neither in this work, nor in that of Mr. Perceval, formerly noticed by us, nor in the reminiscences of Robert Hall, nor in the recent remarkable publication of Mr. Paternoster, do we find any weak partiality for handcuffs, any regretful tears on account of the disuse of leglocks, any sweet associations treasured up with the memory of strait-waistcoats and chains. All the partiality, all the favour and affection, and all the tears, if any be shed, are on the side, not of those who wore the restraints but of those who put them on. If the question related to a mode of punishment, the testimony of the punished might be objected to. The country justice does not permit his reverence for the treadmill to be weakened by hearing that those whom he sends to climb that everlasting stair entertain peculiar objcctions to that mode of exercise. He does not mean it to be a source of pleasure; his singleness of view excludes all ideas but that of punishing the delinquent. The question of restraint is wholly different; it relates to a means that is or ought to be remedial by its action on the mind.

The notes to the poem are numerous and desultory. He must, however, be an ill-tempered reader who would quarrel with what in the composition the author avows to have given him much pleasure. It is curious to see how a strong leaning to certain religious feelings tinges the worthy poet's views of numerous subjects which we believe it in his kind nature to regard with far less severity. If we might venture to address a word of remonstrance and warning at parting, it would be to eschew all that encourages a hostile and bristling state of mind in relation to occupations, recreations, and even opinions, followed, partaken in, or maintained by so many excellent persons as to justify some doubt of their utter unworthiness. Such a state is as unfavorable to the due balance of the mental faculties as it is to that peace of the affections the loss of which is, in such a large majority of cases, the cause of that delicate balance being disturbed. Nor do we think those parts of the poem the most felicitous in which these harsher indications prevail. The muse seems to have looked askance upon them. We would presume to remind the poet of the juster as well as milder breathings of a great predecessor in the poet's art, who, abjuring the ready condemnation of all who differed from him in opinion, makes this a part of his prayer:

"If I am right, thy grace impart,
Still in the right to stay;
If I am wrong, oh! teach my heart
To find that better way."

